# Time trade-off health state utility values for depression: a systematic review and meta-analysis

**DOI:** 10.1007/s11136-022-03253-5

**Published:** 2022-09-30

**Authors:** Péter György Balázs, Dalma Erdősi, Antal Zemplényi, Valentin Brodszky

**Affiliations:** 1 Doctoral School of Business and Management, Corvinus University of Budapest, Budapest, Hungary; 2 Institute of Social and Political Sciences, Department of Health Policy, Corvinus University of Budapest, Budapest, Hungary; 3 Center for Health Technology Assessment and Pharmacoeconomic Research, Faculty of Pharmacy, University of Pécs, Pécs, Hungary; 4 Center for Health Technology Assessment, Semmelweis University, Budapest, Hungary

**Keywords:** Time trade-off, Depression, Systematic review, Meta-analysis, Utility catalog, Vignette comparison

## Abstract

**Purpose:**

This study aims to systematically review the literature on health utility in depression generated by time trade-off (TTO) method and to compare health state vignettes.

**Methods:**

Systematic literature search was conducted following PRISMA guideline in 2020 November (updated in 2022 March) in Pubmed, Web of Science, PsycInfo, and Cochrane Database of Systematic Reviews. Random effect meta-analysis was conducted to pool vignette-based utility values of mild, moderate, and severe depression and to compare the preferences of depressed and nondepressed population.

**Results:**

Overall, 264 records were found, 143 screened by title and abstract after removing duplicates, 18 assessed full text, and 14 original publications included. Majority of the studies (*n* = 9) used conventional TTO method, and most of the studies (*n* = 8) applied 10-year timeframe. Eight studies evaluated self-experienced health (own-current depression). Six studies assessed vignette-based health states of remitted, mild, moderate, and severe depression, half of them applied McSad measure based health description. Altogether, 61 different utility values have been cataloged, mean utility of self-experienced depression states (*n* = 33) ranged between 0.89 (current-own depression) and 0.24 (worst experienced depression). Pooled utility estimates for vignette-based mild, moderate, and severe depression was 0.75, 0.66 and 0.50, respectively. Meta-regression showed that severe depression (β = −0.16) and depressed sample populations (β =  −0.13) significantly decrease vignette-based utility scores.

**Conclusion:**

Our review revealed extent heterogeneity both in TTO methodology and health state vignette development. Patient’s perception of depression health states was worse than healthy respondents.

**Supplementary Information:**

The online version contains supplementary material available at 10.1007/s11136-022-03253-5.

## Introduction

Depression is a common mental disorder worldwide, affecting more than 264 million people of all age groups [1]. It has a wide range of levels of severity and a variable degree of intensity (e.g., major depression, bipolar disorder, affective disorder), and it is characterized by typical physiological and mental symptoms. This disease can cause sleep and eating dysfunctions, impair emotional and cognitive functions, harm individuals’ self-assessments, and disrupt role functions or ordinary behaviors. Depression is regarded as a chronic condition [2] that can cause functional impairment leading to deterioration in health-related quality of life (HRQoL) or decline in subjective perceptions of social, occupational, and health-related well-being [4, 5].

Health economic evaluations frequently apply the notion of the quality-adjusted life year (QALY) to quantify health gains. The notion of QALY consists of two elements: *quality of life*, which is measured by health state utility, and *quantity of life*, which is expressed in terms of life expectancy. One year of full health equals one QALY [6]. The process of calculating the associated utility may employ either direct methods—such as time trade-off (TTO), the standard gamble (SG), the visual analog scale (VAS) or discrete choice experiments—or indirect methods. The indirect utility of respondents can be measured using either generic (e.g., Health Utility Index, SF-6D) or disease-specific (e.g., Hamilton Depression Rating Scale, Patient Health Questionnaire-9) HRQoL questionnaires. Direct utility is obtained by reference to people’s preferences for a given health state. Indirectly evaluated scores can be transformed into utilities using various weights of societal or patient preferences based on the results of the direct utility assessments [3].

The most frequently recommended generic HRQoL instrument for eliciting indirect utility is the EQ-5D questionnaire (EQ-5D-3L and/or EQ-5D-5L) [7], while TTO is a commonly and strongly advised measurement method for eliciting direct utility. Due to its explicit relationship with QALY and taking into account its relative simplicity, the stronger preferences of respondents (as compared to their preference for SG) and better compliance with the theoretical axioms of economic evaluations (as compared to the measurements of VAS), TTO has become very popular among direct health state preference elicitation techniques over the past 30 years [8].

The time trade-off task is designed to force a respondent to express indifference between living for a period of time ‘*t*’ in a better health state and living for a period of time ‘*x*’ in a particular imperfect health state [9]. Traded years ‘*t*−*x*’ represent the amount (“price”) that the respondent is willing to sacrifice for quality over quantity of life. Utility is calculated directly based on the point of indifference (‘*x*’), at which the preferences of the respondent are equal with respect to the two alternatives. A health utility of ‘1’ equals full health, ‘0’ indicates dead and negative values represent health states that are worse than death (WTD) [10].

For example, in a TTO exercise, the respondent must choose between living 10 years with mild depression or living 9 years with full health, and the utility of mild depression is calculated in terms of the ratio of the indifference point to the length of the time period in question: U = *x*/*t* = 9/10. If 1 year spent in full health equals one QALY, then the outcome of the two alternatives is equalized in terms of the following measure:

$$9{\text{ years }} \times {\text{ utility of full health }}\left( {1.0} \right) = {\text{ }}9{\text{ QALY}} = {\text{ }}10{\text{ years }} \times {\text{ utility of mild depression }}\left( {0.9} \right)$$

TTO features a diverse methodology; the relevant timeframe, iteration process, smallest tradable amount, assessed health state (current-own/self-experienced vs. vignette-based/hypothetical health state), numbers, and orders of evaluated health states, and methods of data collection can vary. Researchers must compromise between adjusting this method to the specific attributes of their studies and/or following a standard protocol to ensure the comparability of results [11]. Many studies have used conventional, composite, or indifference in one answer methods of TTO [12, 13], but alternative forms such as waiting or sleep trade-offs have also appeared in certain papers [14]. Following conventional, composite, or other methodological protocols has a crucial impact on the resulting utilities [12, 15, 16]. Timeframe differences [17], valuing vignette-based vs. self-experienced health states [18, 19], the responding population [20, 21], the health state description system [22], the iteration process [23], and the anchor health state at ‘utility = 1’ [24, 25] can all alter the resulting utilities. It is also important to note that, in addition to methodological attributes, the vignettes that are used to describe the disease and the clinical assessment tools employed can differ significantly.

Health state vignettes describe a given health state in the context of TTO tasks. A description of, for example, ‘mild depression’ may differ in terms of disease domains and severity across vignettes [26, 27]. The aim of health state vignettes is to depict the disease as precisely as possible. Domains describe disease-specific burdens and attributes while simultaneously differentiating the stages of disease severity [28]. Health descriptions are extremely important to obtain accurate utility results, and differences in similar health state descriptions are possible sources of systematic differences in responses [29, 30]. Several studies have reported that presentation of the valuation task has an impact on the values, thus, elicited [31–33]. The development of health state vignettes can employ different practices. Typically, vignettes are based on a literature review/scoping or consultation with (health) professionals. Recent reviews have reported controversial conclusions regarding the impact of vignettes on utility estimates [34, 35].

Compared to a large number of empirical HRQoL outcome studies that have focused on patients with depression, to the best of our knowledge, only two systematic reviews summarizing HRQoL outcome studies in the context of depression have been published. Mohiuddin et al. reviewed utilities derived from the EQ-5D and the standard gamble (SG) in the context of unipolar depression, while Brockbank et al. examined studies reporting the effects of treatment of major depressive disorder [36, 37]. Neither of these sets of authors searched directly for studies that applied TTO utility measurements, although they did identify two empirical TTO studies [38, 39]. Different search methods have been used by previous reviews in depression, all of which are distinct from the approach taken by current research. To address this research gap, our study aims to systematically review all original articles that report direct, depression-related utility elicited by the TTO method and to describe the associate (1) study characteristics, (2) vignette development and (3) cataloged utility in the context of depression health. The secondary purpose of this study is to (4) estimate the pooled utilities of depressed and healthy populations with respect to mild, moderate, and severe depression-related health states based on vignettes.

## Methods

### Search strategy

A systematic literature search was conducted in November 2020 following the principles of the Preferred Reporting Items for Systematic Reviews and Meta-Analyses (PRISMA) [40]. The databases searched were PubMed, Web of Science, PsycINFO, and the Cochrane Database of Systematic Reviews. No language or publication date restrictions were employed. The search was updated in March 2022 to ensure that the review was up to date. The selected keywords were discussed by the authors. The search strategy was developed as a combination of the following terminological variants: ‘time trade-off,’ ‘time tradeoff,’ ‘time trade off,’ ‘TTO,’ and ‘depression’ (for the detailed search strategy, see Supplementary Material 1). Citation tracking of the eligible studies was conducted by searching reference lists by hand.

### Study selection

After removing duplicate studies, two independent reviewers (PB, DE) screened the titles and abstracts of selected articles to determine their eligibility for the study (based on the exclusion and inclusion criteria). Disagreements were resolved via joint discussion among the authors (PB, DE, VB). Potentially relevant full-text articles were retrieved and screened in full; nonrelevant studies were excluded based on eight exclusion criteria.

The exclusion criteria for the title and abstract screening were as follows, listed in hierarchical order: no English abstract, English abstract of non-English full-text paper, not a journal article (e.g., abstract, editorial, letter to the editor), animal/in vitro/in silico or other preclinical study, abstract unrelated to the relevant disease (depression), abstract was a review/secondary search, EQ-5D valuation studies, use of TTO to elicit utility for health states described by EQ-5D descriptive system. The included articles were (1) empirical studies, (2) primer TTO studies focused on depression or depressed states as evaluated via TTO, and (3) studies focused on samples of patients, health professionals, or a general or combined population.

### Data extraction

This review summarizes general information regarding the articles, such as their authors, years of publication, study settings, countries, and main study objectives. Furthermore, the following information was extracted: (1) study population; (2) sample size; (3) proportion of women; (4) age; (5) data collection method; (6) TTO method; (7) time frame; (8) evaluated health state; (9) number of vignettes; (10) description of health states; (11) type of vignette used; (12) number of health-description domains; and (13) health state utility (mean and SD, if available). All included full-text articles were distributed equally between PB and DE, who independently extracted data from the articles. Unclear cases were resolved via joint discussion. Considering that there is no standard quality assessment protocol for TTO measurements and the age of the included studies, we used seven aspects of the earlier EuroQol valuation protocol, the Measurement and Valuation of Health (MVH) to evaluate the TTO studies: framework, time horizon, anchor state, iteration algorithm, mode of administration, method of data collection and respondent training [41].

### Statistical analysis

Meta-analysis was conducted using the random effect (REML) model to evaluate mean utility estimates. By default, two eligibility criteria were established. The pooled utility of (1) mild, moderate, and severe depression (2) described by vignettes was included in the meta-analysis, ensuring that comparisons were made only between vignette-based health states. The missing standard deviation (SD) data were replaced by the sample size-weighted average ($\bar{u} = \frac{{\sum {u_{i} } \times n_{i} }}{{\Sigma n_{i} }}$) of reported SDs [42]. The average utility of three vignette-based health states—mild, moderate, and severe depression—were compared across depressed and nondepressed populations. The effects of four binary coded variables, i.e., *vignette type* (McSad or other); *population group* (depressed or nondepressed); *method* of *data collection* (self-completed or interviewer-administered); and *depression severity* (mild or severe), on utility were analyzed via a meta-regression. Heterogeneity was tested using I^2^, which measures the proportion of observed variation between studies, in which context the differences were expressed in terms of utility estimates (0–100%). The dispersion between studies (variance of utilities) was estimated by computing T^2^ and Tau [43]. The meta-analysis forest plots and the meta-regression were developed using Stata 16.0 software (StataCorp LLC).

## Results

### Study selection

**Fig. 1:**
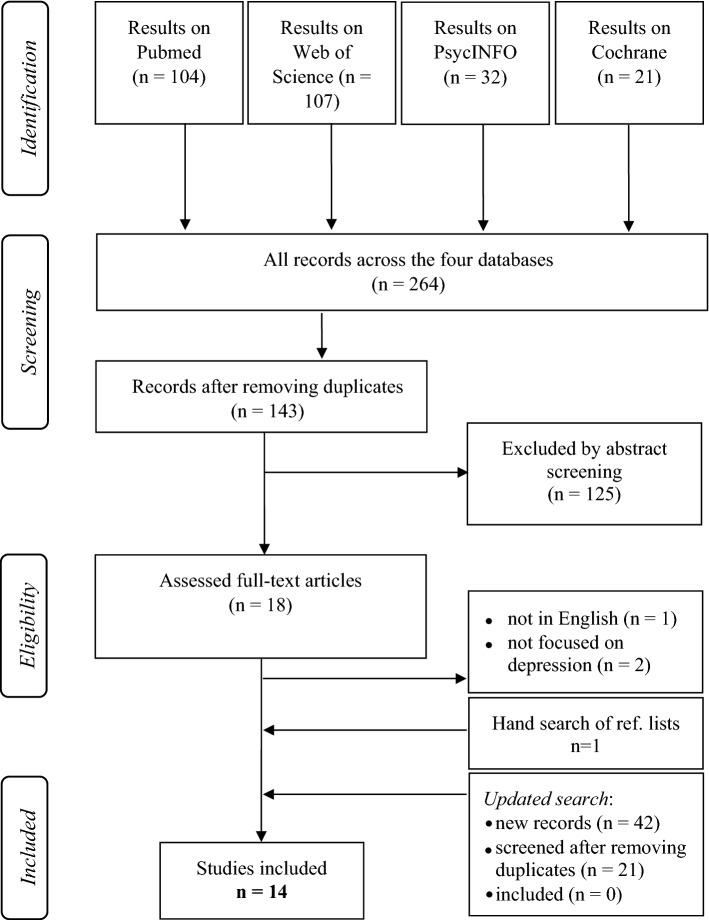
Prisma flowchart of the literature search process

Overall, 264 records were found across four databases (PubMed: 104; Web of Science: 107; PsycINFO: 32; Cochrane: 21). After duplicates were removed (*n* = 121), the abstracts and titles of 143 articles were screened, of which 125 articles were excluded. Diseases/health states that were unrelated to depression and EQ-5D evaluation studies were the most common reasons for such exclusion. Accordingly, 18 articles were included for full-text analysis, due to which an additional 4 articles were discarded (not in English = 1 [44]; not focused on depression = 2 [45, 46]; design for an unrealized trial = 1 [47]. In total, 14 articles met the inclusion criteria [38, 48–58]. Two publications from one study [59, 60] were merged based on the use of the same sample population and TTO method. One additional empirical TTO publication was found by hand searching the reference lists, resulting in 14 included studies. The updated search found 42 new records (PubMed: 121; Web of Science: 124; PsycINFO: 39; Cochrane: 22), and no additional studies eligible for inclusion were found (Fig. 1).

### Study characteristics

The studies were heterogeneous in terms of study setting, patient characteristics, health status, data collection, and description of health status vignettes (Table 1). All studies were published between 1991 and 2020, and they were conducted in 9 countries: 4 in the US [39, 48, 49, 56], 2 in Canada [50, 59, 60], 2 in the Netherlands [54, 55] and one each in the United Kingdom, Thailand [58], Australia [51], Sweden [38], Spain [53] and Germany [52]. The majority of the studies included patients with depression (*n* = 9; 64%)[39, 48–50, 52, 53, 56, 58–60]. Three studies (*N* = 3; 21%) included the general population [38, 54, 55], one study focused on the utilities of health professionals [51] and one study focused on a mixed populations of patients, i.e., the general population and health professionals [57]. The applied study designs were cross-sectional studies (*N* = 11, 79%) [38, 48, 49, 51–58], randomized controlled trials (*N* = 2, 14%) [39, 59, 60], and case control studies (*N* = 1, 7%) [50]. The most frequently used data collection methods were semistructured interviews (*N* = 8, 57%) [48–50, 52, 54, 58–60], paper-based self-completion questionnaires (*N* = 4, 29%) [38, 39, 51, 53] and online self-completed questionnaires (*N* = 2, 14%) [55, 57]. The sample sizes varied widely between 32 and 3986; similarly, the mean age of respondents varied between 32.0 and 52.8 years (two studies reported only the minimum and maximum age: 20–64 years), and the proportions of women varied between 11.5–93.5%.

**Table 1:** Study characteristics

Publication	Study setting	Main objective	Country	Study population	Data collection	Sample size	Women %	Age (mean or range)
**Self-experienced health valuing TTO studies **								
Oldridge et al. [59, 60]	Randomized controlled trial	Evaluating clinical effectiveness of rehabilitation	Canada	Mildly/moderately depressed patients diagnosed with myocardial infarction	Semi-structured interviewer administrated	165	11.5%	52.8
Wells and Sherbourne [48]	Cross-sectional	Compare both HrQoL and utility for current health	USA	Self-reported (on DSM-IV) depression outpatients	Semi-structured interviewer administrated	750	62.0%	NR
Tsevat at al. [49]	Cross-sectional	Measure health values of patients with bipolar disorder	USA	Treatment receiving bipolar disorder patients	Semi-structured interviewer administrated	53	62.0%	43
Voruganti et al. [50]	Case-control	Explore the feasibility of traditional utility evaluation techniques	Canada	Diagnosed major depression patients (on DSM-IV)	Semi-structured interviewer administrated	32	53.3%	43.6
Sherbourne et al. [39]	Randomized controlled trial	Evaluating the responsiveness of seven HrQoL measures	USA	Clinician assessed patients with depressive symptoms	Paper based—self-completed	1136	71.0%	44.3
Isacson et al. [38]	Cross-sectional	Measuring the impact of depression on HrQoL	Sweden	General population with self-reported depression	Paper based—self-completed	3986	53.7%	20-64
König et al. [52]	Cross-sectional	Analyze the TTO method properties in patients with mental disorders	Germany	Patients diagnosed with affective disorder (ICD-10)	Semi-structured interviewer administrated	153	66.7%	46.8
Leykin et al. [56]	Cross-sectional	Compare preferences of depression patients with comorbid patient groups	USA	Major depressive disorder patients diagnosed via DSM-IV	Semi-structured interviewer administrated	61 MDD patients	54.1% MDD patients	40.1 MDD patients
						58 MDD+pain comorbid p.	60.3% MDD+pain comorbid p.	50.9 MDD+pain comorbid p.
**Vignette-based depression states valuing TTO studies**								
Sanderson et al. [51]	Cross-sectional pilot study	Modeling changes in health status	Australia	Health professionals: general practitioners	Paper based—self-completed	42	63.0%	35-54
Montejo et al. [53]	Cross-sectional	Multi-attribute utility (MAU) tool development	Spain	Bipolar disorder and schizophrenia patients (according to DSM-IV)	Paper based—self-completed	70	36.2%	41.9
Papageorgiu et al. [54 ]	Cross-sectional pilot study	Pilot test TTO valuation task and vignettes	Netherlands	Quota sampled volunteers of general population	Semi-structured interviewer administrated	60	50.0%	35.0
Papageorgiu et al. [55]	Cross-sectional	Direct utility elicitation with TTO in vignette-based depression health states	Netherlands	Stratified sampling of general population	Online—self-completed	NR	51.1%	46.7
Flood et al. [57]	Cross-sectional	Develop and assess health state vignettes and TTO task	United Kingdom	Volunteers among mental service users, health professionals and caregivers	Online—self-completed	46 patients	73.9%	32.0
						31 general population	93.5%	36.0
						28 health profession.	67.9%	39.0
Nontarak et al. [58]	Cross-sectional	Determine disability weights for depression	Thailand	Diagnosed major depressive disorder patients (ICD-11: F32)	Semi-structured interviewer administrated	75	69.3%	47.9

*MDD* major depressive disorder

### TTO methodological attributes

The vast majority of studies employed the conventional time trade-off method (*N* = 9; 64%) [38, 48–50, 54, 56–60], three studies (21%) used an indifference in one answer task [39, 51, 55], one study (7%) applied lead-time/lag-time TTO [52], and one paper did not report its protocol [53]. Overall, eight studies used a 10-year timeframe [39, 48, 51, 55–60], and six individual studies used various time frames: 10 years + y years lead/lag time[52], 20 years [38], 50 years [50], 80-year-old ages [54], subjective life expectancy [49], and alternating periods of time (among 20, 25, 30, 35, and 40 years) [53]. The process of iteration was poorly reported (*N* = 6 missing) [38, 48–50, 53, 59, 60]. Three studies used a single question [39, 51, 55], two studies used the incremental bottom-up method [57, 58], another two studies used the ping-pong method [54, 56], and one study employed top-down steps [52]. Self-experienced health (affective disorder/bipolar disorder/depression/depressive symptoms/depression following infarction) was assessed by seven studies [38, 39, 48–50, 52, 59, 60], vignette-based health (no, mild, moderate, and severe depression) was focused on by six studies [51, 53–55, 57, 58], and one study [56] evaluated perfect health vs. self-experienced health vs. mild depression. All but one study limited its focus to the better than death (BTD) format, while König et al. [52] complemented this approach with a lead/lag-time TTO task, which could yield utilities ranging from negative values to 1 (Table 2). The TTO study quality met the requirements of MVH protocol mostly in attributes of framework (79%) and timeframe (64%), the detailed process of iteration and respondent training (50% and 57% missing) were rather poorly reported. (Supplementary material 3).

**Table 2:** Time trade-off task methodological attributes

Publication	TTO type	Timeframe	Iteration process	Health state description	Format
**Self-experienced health valuing TTO studies**					
Oldridge et al. [59, 60]	conventional	10 years	NR	current health	BTD
Wells and Sherbourne [48]	conventional	10 years	NR	current health	BTD
Tsevat at al. [49]	conventional	subjective life expectancy	NR	current mental health	BTD
Voruganti et al. [50]	conventional	50 years	NR	current mental health, worst state mental health, desirable mental health	BTD
Sherbourne et al. [39]	indifference in one answer	10 years	single question	current health	BTD
Isacson et al. [38]	conventional	20 years	NR	current health	BTD
König et al. [52]	conventional	10 years +10 years waiting	top-down steps	current health	BTD+waiting trade-off
Leykin et al. [56]	conventional	10 years	ping-pong method (7 years basepoint)	perfect health vs current health and current health vs mild depression	BTD
**Vignette-based** **depression states valuing TTO studies**					
Sanderson et al. [51]	indifference in one answer	10 years	single question	vignettes: remitted, few symptom, some symptom, many symptom depression	BTD
Montejo et al. [53]	NR	20, 25, 30, 35, 40 years	NR	vignette: single statement	BTD
Papageorgiu et al. [54]	conventional	80-year-old age	ping-pong method	vignettes: mild and severe depression + co-occurring with cancer, diabetes, heart disease (2+6)	BTD
Papageorgiu et al. [55]	indifference in one answer	10 years	single question (ping-pong method as warm up)	vignettes: no, mild, moderate, severe depression	BTD
Flood et al. [57]	conventional	10 years	incremental (bottom-up)	vignettes: severe depression	BTD
Nontarak et al. [58]	conventional	10 years	top-down steps	vignettes: mild, moderate, severe depression	BTD

*BTD* better than dead health state; *NR* not reported

### Health state vignettes

The comparison of the reviewed vignettes and their characteristics are summarized in Table 3. Overall, six studies (43%) employed a vignette-based TTO task [51, 53–55, 57, 58] describing remitted, mild, moderate, and severe states of depression. The vignettes covered 11 dimensions: emotions, physiology, mood, anxiety, cognition, behaviors, role function, social relations, usual activities, and self-appraisal. The number of dimensions used in the studies ranged from 1 to 6, with a mode of six. The number of evaluated health states ranged between 1 and 8. Two studies (33%) evaluated mild, moderate, and severe depression [55, 58]; one study (16%) evaluated no/in-remission, mild, moderate, and severe states of depression [51]; two studies investigated only the severe level of depression [53, 57]; and one study evaluated mild and severe depression separately alongside three co-occurring diseases (cancer, diabetes, and heart disease) [54]. The number of designed vignettes included in these studies ranged from 1 to 30. Almost all studies (83%) used one vignette for each different level of depression severity [51, 53, 54, 57, 58], while one study [55] designed 4 mild, 17 moderate and 9 severe vignettes to differentiate vignette-based depression-related health states. Regarding the presentation of vignette-based health states, three studies (50%) used scenarios (which were interpreted from a third-person perspective) [51, 54, 55], while three studies (50%) used statements as descriptions [53, 57, 58].

**Table 3:** Health state vignette comparison

Publication	Year	Dimensions	Number of dimensions	Health states	Number of health states assessed	Origin of health state description	Vignettes in the study	Presentation of health state description
Sanderson et al.	2004	emotions, functioning, mood, physiology, cognition, social relations	6	remitted, few symptoms, some symptoms, many symptoms	4	SF-12 questionnaire	4	statements
Montejo et al.	2011	anxiety/depression	1	severe depression	1	TooL questionnaire	1	single statement
Papageorgiou et al.	2014	emotion, self-appraisal, cognition, physiology, behavior, role function	6	mild/severe depression & mild depression co-occurring with cancer/diabetes/heart diseases	2+6	McSad depression scale	8	statements
Papageorgiou et al.	2015	emotion, self-appraisal, cognition, physiology, behavior, role function	6	mild, moderate, severe	4	McSad depression scale	30	statements
Flood et al.	2017	self-appraisal, physiology, functioning, emotions, social relations, usual activities	6	severe depression	1	McSad depression scale	1	scenario
Nontarak et al.	2020	emotion, usual activities, physiology, cognition	5	mild, moderate, severe	3	NR	3	scenario

Attributes of disease were covered in a similar manner: dimension items focused on emotions, physiological functioning, and cognition appeared in five out of six descriptions. Only one research group used the same vignette design in two studies [54, 55]; descriptions notably differed across vignettes. Three of six descriptions used the McSad depression scale (which was originally developed for direct utility measurement), although the method of presentation differed across cases (statements vs. scenario) [54, 55, 57]. One study used 6 items of the SF-12 (MCS), which were presented as statements, and altered the original response options to describe 4 levels of depression severity [51]. Another study focused on a single dimension, single statement description, using the first item of the mental health-specific Tolerability and Quality of Life (TooL) questionnaire [53]. The basis of the description design was not disclosed by one study; however, the description closely resembled the scenario-based McSad vignette [58].

### Health state utilities

Overall, 61 utilities were extracted from 14 original studies. Nine studies (64%) [39, 48–50, 52, 53, 56, 58–60] reported the utilities of depression patients (physician or self-diagnosed), two studies focused on the nondepressed population (general population, health professionals), [51, 54] and three emphasized both the depressed and the nondepressed population [38, 55, 57]. Supplementary Material 2 contains the utility catalog, which indicates the descriptions of the included study populations alongside the health states and utilities examined (means, SDs).

Eight studies (57%) [38, 39, 48–50, 52, 56, 59, 60] calculated 36 utilities pertaining to 33 different self-experienced depression states among respondents, ranging from 0.89 (self-experienced health state of US depression patients) to 0.24 (worst own health state experienced by Canadian depression patients). Six studies (43%) [51, 53–55, 57, 58] calculated 25 vignette-based utilities for no, mild, moderate, and severe levels of depression as well as six comorbid conditions co-occurring with depression. These values ranged from 0.96 (reported depression as evaluated by health professionals) to 0.31 (patient perceptions of severe depression resulting from mental illness).

### Results of the meta-analysis

Our meta-analysis examined three different vignette-based depression-related health states (mild: *n* = 5; moderate: *n* = 4; severe: *n* = 9) derived from patients (*n* = 3), the general population of depressed/nondepressed persons (*n* = 4) and health professionals (*n* = 2). Utility estimates were pooled and compared between the study populations without depression (*n* = 11) and with depression (*n* = 7).

**Fig. 2:**
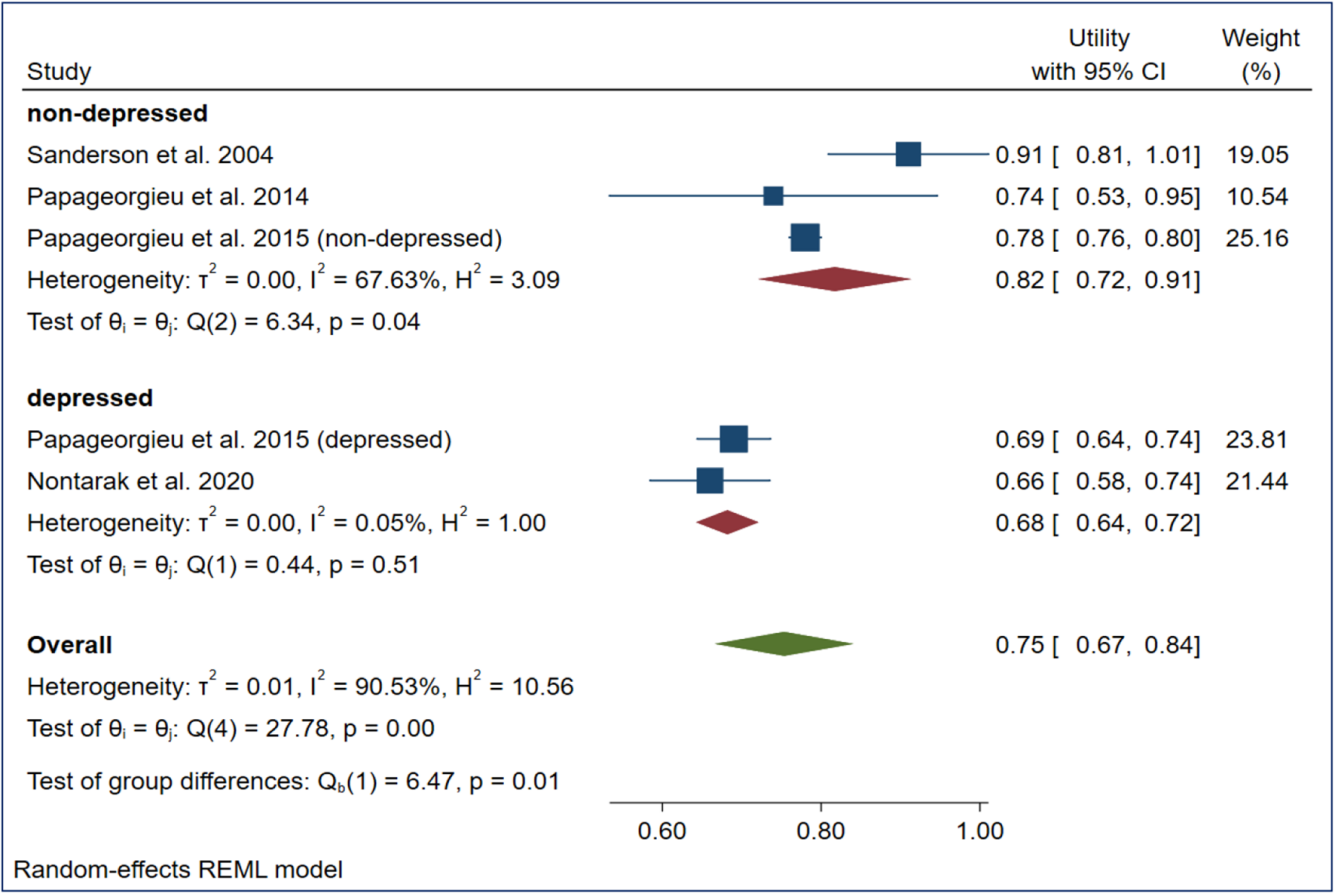
Meta-analysis of utility estimates in vignette-based mild depression (forest plot)

**Fig. 3:**
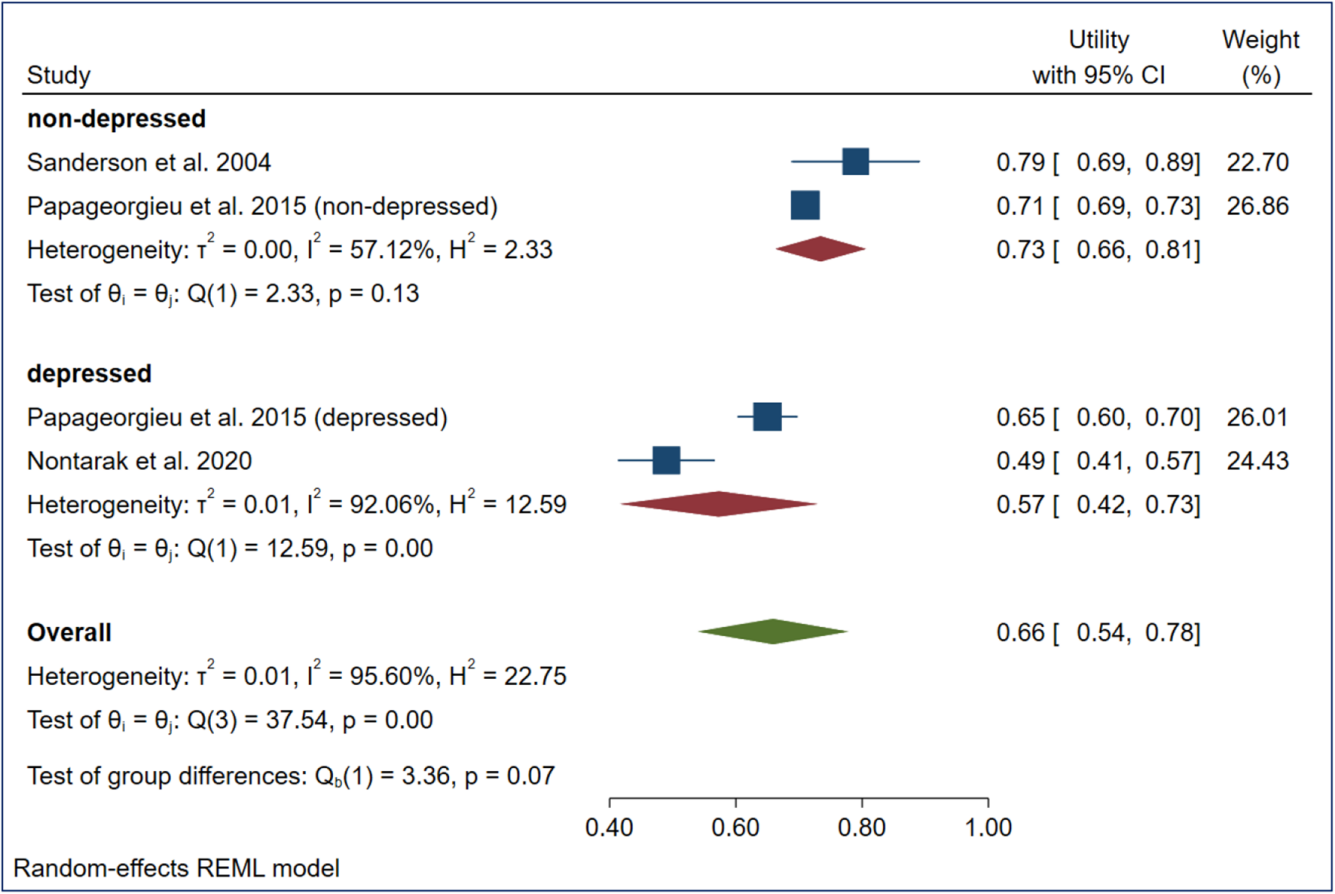
Meta-analysis of utility estimates in vignette-based moderate depression (forest plot)

**Fig. 4:**
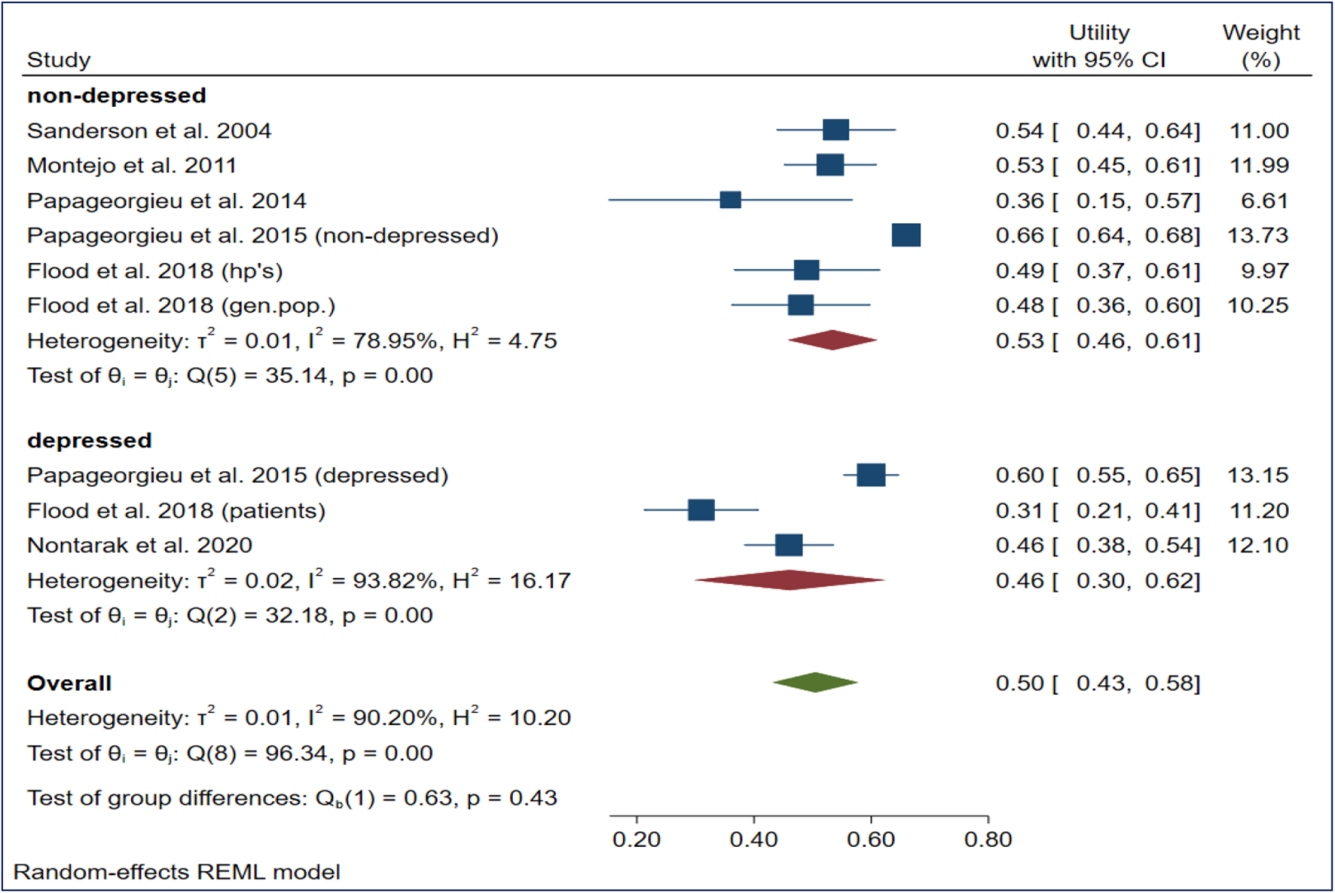
Meta-analysis of utility estimates in vignette-based severe depression (forest plot)

The estimated mean utilities (upper-lower confidence level of 95%) of vignette-based mild, moderate, and severe depression values were 0.82 (0.72–0.91), 0.73 (0.66–0.81), and 0.53 (0.46–0.61) in the nondepressed population and 0.68 (0.64–0.72), 0.57 (0.42–0.73), and 0.46 (0.30–0.63) in the depressed population, respectively (Figs. 2, 3, 4). The overall mean TTO utilities were 0.75 in mild depression, 0.66 in moderate depression and 0.50 in severe depression (See Figs. 2, 3, 4).

A large proportion of I^2^ was observed among the pooled utilities of the moderate (92.1%) and severe (93.8%) depression subgroups. All subgroups exhibited a low (standard) deviation of utilities across studies (T^2^ = 0.000–0.020). The meta-regression showed that evaluating a severe depression state (β = − 0.16) and focusing on a depressed population sample (β = − 0.13) had small but significant (*p* < 0.05) negative effects on the resulting utilities (Table 4).

**Table 4:** Meta-regression results of four variables on utility estimates

Random effect meta-regression				
Moderator		Coefficient	S.E.	P
Depression (reference coded: moderate depression)	Mild	0.088	0.065	0.178
	Severe	*− 0.155*	0.062	*0.012*
Vignette type	McSad based (ref. category: other)	0.016	0.053	0.756
Data collection	Interview (ref. category: self-completed)	0.037	0.063	0.552
Population sample	Depressed population (ref. category nondepressed)	*− 0.128*	0.054	*0.017*
Residual I²	88.80%			
I²	97.50%			
Residual T²	0.0008			
T²	0.0210			

Italic values indicate the explanatory variables that significantly impact pooled vignette-based utility estimates

## Discussion

In our review, all empirical studies reporting vignette-based or self-experienced TTO utility in the context of depression were collected to produce a catalog of reported utilities and compare vignettes concerning health states. The pooled mean utilities of different depression-related health states elicited in both patients and healthy individuals were estimated in a meta-analysis.

Our review identified 14 articles reporting 36 self-experienced and 25 vignette-based utilities for depression-related health states. The utilities reported by two randomized control trials (measuring the effects of usual care vs. rehabilitation and enhanced psychotherapy & management care) [39, 59, 60] accounted for one third of the 61 health state utilities cataloged. The mean utility of depression patients’ self-experienced health ranged between 0.89 and 0.24, while the vignette-based mean utility of mild, moderate, and severe depression ranged between 0.91–0.66, 0.79–0.49 and 0.66–0.31, respectively. For comparison, previously reviewed SG and EQ-5D utilities in the context of unipolar depression ranged from 0.92–0.09 to 0.90–0.14, respectively [36].

Currently, little is known regarding the comprehensive impact of various health state vignettes on the elicited TTO utilities. Vignette designs are not standardized and heterogeneous in terms of the domains they cover, their modes of presentation and their origins, although methodological recommendations are provided [61] to guide future researchers. Six vignette-based studies were included in our review, which employed five different methodological approaches to vignette development. The description of health states was based on the McSad depression scale in three studies. Our review showed that nonstandardized vignette development resulted in different health state vignettes even when the same standard scale was employed. Our findings support the use of a common approach to vignette development [26, 29, 61].

The overall pooled vignette-based TTO utilities for mild, moderate, and severe depression states were 0.75, 0.66 and 0.50, respectively. Meta-regression revealed the significant negative impact of severe depression (β = − 0.155) and the depressed population (β = − 0.128) on these pooled utility estimates. The high proportion of heterogeneity found in this case suggests the existence of additional subgroup or moderator effects, especially in cases of severe and mild depression. Our pooled TTO utilities were higher than the SG and EQ-5D utilities reported in a previous meta-analysis of studies focusing on patients with unipolar depression (mild: 0.75, 0.69 and 0.56, moderate: 0.66, 0.52 and 0.45, severe: 0.50, 0.27 and 0.25) [36]. Our findings support the claim that patients report different health utilities than the general population [22, 62]. Many studies have suggested that patients generally have higher utilities that are attributable mostly to experience-based evaluations [63, 64]. Our study contradicts the assumption that patients report higher utilities; in all comparable (*N* = 18) vignette-based health states, depressed groups had lower pooled mean utility in cases of mild, moderate, and severe depression.

Remarkable differences between various methods of utility generation have been reported [65–67]. Nine of the included studies used methods other than TTO to elicit utilities pertaining to the same health state. Three studies [49, 50, 57] compared rating scale (RS) vs. SG vs. TTO and reported a consistent order of self-experienced depression utilities: RS < TTO < SG. The claim that RS < TTO utility was further supported by a vignette-based assessment [51]. Another three studies compared the valuations of SG and TTO, in which context SG indicated higher utility in all cases [39, 48, 56]. Vignette-based evaluation of mild, moderate, and severe depression comparing 3 × 3 mean utilities indicates an order of EQ-5D < TTO < VAS, with the three approaches exhibiting considerable differences [58]. In the context of affective disorder, [52] German value set-based utility (0.79) was higher than TTO (0.66), but UK value set-based EQ-5D-3 L utility was lower (0.63). This review suggests that the utility generation method may significantly impact the utility value associated with depression.

This study faces certain limitations. First, the substantial heterogeneity of the included studies made it difficult to compare utilities across studies and to analyze the impact of TTO task attributes on utility values. Similarly, the heterogeneity of the studies included in the meta-analysis and the differently described vignette-based health states should be noted. Second, TTO study quality evaluation included as supplementary information was based on MVH protocol for TTO valuations, which was advanced by the EuroQol’s EQ-VT valuation protocol in 2012. However, majority of the papers were published earlier, the MVH protocol was the most suitable quality check for TTO studies [14, 41, 68]. Third, we included only English-language publications, which may have led to the exclusion of studies relevant to the research question.

## Conclusions

Despite the wide range of empirical studies that have examined HRQoL using generic or disease-specific tools to examine utility in the context of depression, [3, 69, 70] studies measuring health state via the time trade-off method have not hitherto been reviewed. To our knowledge, this study is the first to compare health state vignettes in the context of depression and to provide a comprehensive catalog of TTO utility. Our review revealed the extent of heterogeneity both in TTO methodology and in the development of health state vignettes. The meta-regression showed that a severe level of depression and the inclusion of a depressed sample decreases utility. Interestingly, in contrast with the findings of previous TTO reviews [63, 71], patients’ perceptions of depression-related health states were worse than those of healthy respondents.

## Supplementary Information

Below is the link to the electronic supplementary material.

Supplementary file1 (DOCX 15 kb)

Supplementary file2 (DOCX 23 kb)

Supplementary file3 (DOCX 18 kb)
