# Supplementary material for: Time trade-off health state utility values for depression: a systematic review and meta-analysis
Source: Qual Life Res. 2022 Sep 30;32(4):923–37. doi: 10.1007/s11136-022-03253-5 (PMC10063515; doi:10.1007/s11136-022-03253-5)
Supplement: Supplementary file 1 — Supplementary file1 (DOCX 15 kb) [file 11136_2022_3253_MOESM1_ESM.docx]

| *#* | *search terms* | *result: Pubmed* | *result: Web of Science* | *result: Cochraine* | *result: PsycInfo* |
| --- | --- | --- | --- | --- | --- |
| #1 | "time tradeoff"[All Fields] OR "time trade-off"[All Fields] OR "time trade-off"[All Fields] OR "TTO"[All Fields] | 2,223 | 3,427 | 292 | 461 |
| #2 | "depressed"[All Fields] OR "depression"[MeSH Terms] OR "depression"[All Fields] OR "depressions"[All Fields] OR "depression's"[All Fields] OR "depressive disorder"[MeSH Terms] OR ("depressive"[All Fields] AND "disorder"[All Fields]) OR "depressive disorder"[All Fields] OR "depressivity"[All Fields] OR "depressive"[All Fields] OR "depressively"[All Fields] OR "depressiveness"[All Fields] OR "depressives"[All Fields] | 567,587 | 581,299 | 87,719 | 376,874 |
| #3 | #1 AND #2 | 121 | 124 | 22 | 39 |

**Search strategy and results in four databases:**
