# Supplementary material for: Time trade-off health state utility values for depression: a systematic review and meta-analysis
Source: Qual Life Res. 2022 Sep 30;32(4):923–37. doi: 10.1007/s11136-022-03253-5 (PMC10063515; doi:10.1007/s11136-022-03253-5)
Supplement: Supplementary file 2 — Supplementary file2 (DOCX 23 kb) [file 11136_2022_3253_MOESM2_ESM.docx]

Additional file 3 (Utility catalogue)

| **Publication** | **Study population** | **Health state** | **n** | **Utility mean (SD)** |
| --- | --- | --- | --- | --- |
| Oldridge et al. 1991 and 1993 | depression after myocardial infarction patients-rehabilitation | (baseline) self-experienced health state | 78 | 0.72 (0.24) |
|  |  | 2 month follow up | 78 | 0.80 |
|  |  | 4 month follow up | 78 | 0.82 |
|  |  | 8 month follow up | 78 | 0.87 |
|  |  | 12 month follow up | 78 | 0.87 |
|  | depression after myocardial infarction patients-conventional therapy | (baseline) self-experienced health state | 87 | 0.77 (0.25) |
|  |  | 2 month follow up | 87 | 0.82 |
|  |  | 4 month follow up | 87 | 0.82 |
|  |  | 8 month follow up | 87 | 0.83 |
|  |  | 12 month follow up | 87 | 0.86 |
| Wells et al. 1999 | primary care depression patients | self-experienced health state (probable depression) | 750 | 0.89 (0.14) |
|  |  | self-experienced health state (depression + comorbid medical condition) | 2146 | 0.87 (0.13) |
| Tsevat et al. 2000 | patients with bipolar disorders | self-experienced health state (depression) | 53 | 0.71 (0.37) |
| Voruganti et al. 2000 | depression patients | desired health state in the future | 30 | 1.00 |
|  |  | self-experienced (current) health state | 30 | 0.73 (0.19) |
|  |  | worst health state experienced | 30 | 0.24 (0.02) |
| Sherbourne et al. 2001 | patients with depressive symptoms | (baseline) self-experienced health state (depression) | 1018 | 0.83 (0.31) |
|  | Usual care received patients | 6 month follow up | 372 | 0.80 |
|  |  | 12 month follow up |  | 0.81 |
|  |  | 18 month follow up |  | 0.84 |
|  |  | 24 month follow up |  | 0.86 |
|  | Quality improved management received patients | 6 month follow up | 361 | 0.79 |
|  |  | 12 month follow up |  | 0.81 |
|  |  | 18 month follow up |  | 0.84 |
|  |  | 24 month follow up |  | 0.86 |
|  | Quality improved psychotherapy received patients | 6 month follow up | 403 | 0.76 |
|  |  | 12 month follow up |  | 0.80 |
|  |  | 18 month follow up |  | 0.83 |
|  |  | 24 month follow up |  | 0.87 |
| Sanderson et al. 2004 | health professionals (GP's) | remitted depression | 42 | 0.96* |
|  |  | few symptom-depression | 42 | 0.91 |
|  |  | some symptom-depression | 42 | 0.79 |
|  |  | many symptom-depression | 42 | 0.54 |
| Isacson et al. 2005 | general population | self-experienced health state | 3835 | 0.93 (0.15) |
|  | general population (self-reported depression) |  | 151 | 0.80 (0.25) |
| König et al. 2009 | patients | affective disorder | 153 | 0.66 (0.44) |
| Montejo et al. 2011 | schizophrenia and bipolar disorder patients | severe depression /anxiety | 70 | 0.53 |
| Papageorgiu et al. 2014 | general population | mild depression | 10 | 0.74 |
|  |  | severe depression | 10 | 0.36 |
|  |  | mild depression-cooccurring with moderate cancer | 7 | 0.65 |
|  |  | mild depression-cooccurring with moderate diabetes | 6 | 0.63 |
|  |  | mild depression-cooccurring with moderate heart disease | 7 | 0.63 |
|  |  | mild depression-cooccurring with severe cancer | 7 | 0.45 |
|  |  | mild depression-cooccurring with severe diabetes | 6 | 0.47 |
|  |  | mild depression-cooccurring with severe heart disease | 7 | 0.59 |
| Papageorgiu et al. 2015 | general population - depressed | mild depression | 200 | 0.69 |
|  |  | moderate depression |  | 0.65 |
|  |  | severe depression |  | 0.60 |
|  | general population - non depressed | mild depression | 1068 | 0.78 |
|  |  | moderate depression |  | 0.71 |
|  |  | severe depression |  | 0.66 |
| Leykin et al. 2017 | depression patients | self-experienced (own) depression-compared to perfect health | 61 | 0.46 (0.31) |
|  |  | own depression-compared to mild depression | 61 | 0.79 (0.34) |
|  | comorbid patients (depression+pain) | self-experienced (own) depression-compared to perfect health | 58 | 0.52 (0.34) |
|  |  | self-experienced (own) depression-compared to mild depression | 58 | 0.81 (0.30) |
| Flood et al. 2018 | mental illness patients | severe depression | 46 | 0.31 (0.35) |
|  | general population |  | 31 | 0.48 (0.30) |
|  | health professionals |  | 28 | 0.49 (0.34) |
| Nontarak et al. 2020 | major depressive disorder patients | mild depression | 75 | 0.66 |
|  |  | moderate depression | 75 | 0.49 |
|  |  | severe depression | 75 | 0.46 |
