# Supplementary material for: Time trade-off health state utility values for depression: a systematic review and meta-analysis
Source: Qual Life Res. 2022 Sep 30;32(4):923–37. doi: 10.1007/s11136-022-03253-5 (PMC10063515; doi:10.1007/s11136-022-03253-5)
Supplement: Supplementary file 3 — Supplementary file3 (DOCX 18 kb) [file 11136_2022_3253_MOESM3_ESM.docx]

Assessments of included studies according to EQ-5D valuation protocols

| **Study #** | **Protocol** | **TTO framework** | **Time horizon** | **Anchor state** | **Iteration algorithm** | **Mode of administration** | **Method of data administration** | **Respondent training** | **Score** |
| --- | --- | --- | --- | --- | --- | --- | --- | --- | --- |
| Oldridge et al. 1991, 1993 | unclear | ✓ | ✓ | ✓ | nr.* | ✓ | nr. | nr. | 4 |
| Wells et al. 1999 | unclear | ✓ | ✓ | - | nr. | ✓ | nr. | nr. | 2 |
| Tsevat at al. 2000 | unclear | ✓ | - | - | nr. | ✓ | ✓ | nr. | 1 |
| Voruganti et al. 2000 | unclear | ✓ | - | ✓ | nr. | ✓ | ✓ | ✓ | 4 |
| Sherbourne et al .2001 | unclear | ✓ | ✓ | - | nr. | - | - | nr. | -1 |
| Sanderson et al. 2003 | unclear | - | ✓ | - | - | - | ✓ | ✓ | -1 |
| Isacson et al. 2005 | unclear | ✓ | - | ✓ | nr. | - | nr. | nr. | 0 |
| König et al. 2009 | MVH | ✓ | ✓ | - | - | ✓ | ✓ | nr. | 2 |
| Montejo et al. 2011 | unclear | nr. | - | nr. | nr. | - | nr. | nr. | -2 |
| Papageorgiu et al. 2014 | MVH | ✓ | - | - | ✓ | ✓ | ✓ | ✓ | 3 |
| Papageorgiu et al. 2015 | MVH | - | ✓ | ✓ | - | ✓ | ✓ | ✓ | 3 |
| Leykin et al. 2017 | unclear | ✓ | ✓ | - | ✓ | ✓ | - | ✓ | 3 |
| Flood et al. 2018 | unclear | ✓ | ✓ | ✓ | - | ✓ | ✓ | ✓ | 5 |
| Nontarak et al. 2020 | unclear | ✓ | ✓ | ✓ | - | ✓ | ✓ | nr. | 4 |

*nr. = not reported

The task quality check is based on MVH (Measurement and Valuation of Health, 1993) protocol, since most studies were conducted before the launch of EQ-VT in 2012.

The MVH protocol has 11 aspects regarding the TTO task, we used 7 that are sufficiently reported among the included studies. The protocol suggests the following standard for TTO tasks: (1) conventional TTO framework is advised, (2) with 10 year timeframe, (3) full health set as anchor state, (4) bisectional titration introducing the best and worst health states, (5) face to face interviewer administrated data collection, (6) paper based data administration using visual aids and (7) starting with warm up task to train the respondent.

A scoring system was set to evaluate the studies quality, +1 point if met the criteria, -1 if not, 0 if the information was not reported.
